# Data Resource Profile: Melbourne Children’s LifeCourse initiative (LifeCourse)

**DOI:** 10.1093/ije/dyac086

**Published:** 2022-05-10

**Authors:** Meredith O’Connor, Margarita Moreno-Betancur, Sharon Goldfeld, Melissa Wake, George Patton, Terence Dwyer, Mimi L K Tang, Richard Saffery, Jeffrey M Craig, Jane Loke, David Burgner, Craig A Olsson, and the LifeCourse Cohort Investigators, Valerie Sung, Emma Sciberras, Sheena Reilly, John W Toumbourou, Kirsten P Perrett, Catherine Marraffa, Angela Guzys, Jennifer J Koplin, Stephanie J Brown, Gehan Roberts, Jon Quach, Tim J Silk, Avihu Boneh, Delyse Hutchinson, Evelyne Muggli, Sharon Lewis

**Affiliations:** Murdoch Children’s Research Institute, Melbourne, Australia; Department of Paediatrics, The University of Melbourne, Melbourne, Australia; Department of Paediatrics, The University of Melbourne, Melbourne, Australia; Clinical Epidemiology and Biostatistics Unit, Murdoch Children’s Research Institute, Melbourne, Australia; Murdoch Children’s Research Institute, Melbourne, Australia; Department of Paediatrics, The University of Melbourne, Melbourne, Australia; Centre for Community Child Health, Royal Children’s Hospital, Melbourne, Australia; Murdoch Children’s Research Institute, Melbourne, Australia; Department of Paediatrics, The University of Melbourne, Melbourne, Australia; Liggins Institute, University of Auckland, Grafton, Auckland, New Zealand; Department of Paediatrics, The University of Melbourne, Melbourne, Australia; Centre for Adolescent Health, Murdoch Children’s Research Institute, Melbourne, Australia; Murdoch Children’s Research Institute, Melbourne, Australia; Department of Paediatrics, The University of Melbourne, Melbourne, Australia; Department of Women’s and Reproductive Health, University of Oxford, Oxford, UK; Murdoch Children’s Research Institute, Melbourne, Australia; Department of Paediatrics, The University of Melbourne, Melbourne, Australia; Department of Allergy and Immunology, Royal Children’s Hospital, Melbourne, Australia; Murdoch Children’s Research Institute, Melbourne, Australia; Department of Paediatrics, The University of Melbourne, Melbourne, Australia; Murdoch Children’s Research Institute, Melbourne, Australia; Department of Paediatrics, The University of Melbourne, Melbourne, Australia; IMPACT—the Institute for Mental and Physical Health and Clinical Translation, School of Medicine, Deakin University, Geelong, Australia; Murdoch Children’s Research Institute, Melbourne, Australia; Department of Paediatrics, The University of Melbourne, Melbourne, Australia; Department of Paediatrics, The University of Melbourne, Melbourne, Australia; Inflammatory Origins Group, Murdoch Children’s Research Institute, Royal Children’s Hospital, Melbourne, Australia; Department of General Medicine, Royal Children’s Hospital, Melbourne, Australia; Department of Pediatrics, Monash University, Melbourne, Australia; Department of Paediatrics, The University of Melbourne, Melbourne, Australia; Centre for Adolescent Health, Murdoch Children’s Research Institute, Melbourne, Australia; Centre for Social and Early Emotional Development, School of Psychology, Faculty of Health, Deakin University, Australia

Key Features

- The Melbourne Children’s LifeCourse Initiative enables researchers to more effectively leverage the value of existing cohorts to improve child and adolescent health.
- The initiative includes 33 studies hosted by or in collaboration with the Murdoch Children’s Research Institute, including 22 core longitudinal cohorts that are fully catalogued.
- These core studies capture health and development for >40 000 children, young people and families tracked for up to four decades, enriched through linkage to administrative data and collection of biosamples.
- Metadata are standardized and curated by LifeCourse to allow researchers internationally to search and browse information about the available data and request data access via https://lifecourse.melbournechildrens.com.
- Beyond the significant contributions of individual studies, a programme of research working across cohorts is increasingly emerging that addresses a shared interest in pathways leading to mental health, cardiometabolic health and immune-related conditions.
- Efforts of cohort researchers are informed by partnering statisticians’ ongoing development and refinement of analytical methods for observational studies, with a key focus on causal inference to inform the development of policy, interventions and trials.

## Data resource basics

The major health challenges facing children and adolescents increasingly reflect chronic problems that have complex biopsychosocial and environmental dimensions.^1^ In high-income countries, nearly a quarter of children and adolescents are overweight or obese,^2^ up to a quarter experience a mental disorder in any given year,^3^ allergic diseases continue to rise^4^ and life-threatening diseases such as cancer remain a major problem.^5^ Overall, children and young people from disadvantaged backgrounds are disproportionately impacted by adverse health and developmental outcomes^6^^,^^7^ and these disparities are expected to be further exacerbated in the context of the COVID-19 pandemic.^8^ Despite their burden and impact on health pathways across the life course,^9^^,^^10^ the origins of many of the major health issues facing children and adolescents are not yet well understood. This limits our capacity to realize opportunities for early prevention and intervention (e.g. on factors such as social disadvantage and infection) that may have cumulative health benefits over the life course.^10^

Cohort studies provide a valuable resource in this regard, particularly when enriched with biospecimens. They allow the impacts of early-life exposures and complex biopsychosocial mechanisms to be investigated over extended periods of development, including those exposures that could never be ethically, cost-effectively or time-efficiently examined within stand-alone randomized trials.^11^ This is facilitated by recent methodological advances, which provide a framework for analysing causal relationships through explicit emulation of the hypothetical ‘target trial’ that would have ideally been conducted.^11^^,^^12^ In addition, there remains a largely untapped potential to work systematically across cohorts. For example, appropriate harmonization and pooling of data from multiple cohorts can improve precision of estimation when investigating rare exposures and/or outcomes.^13^ Cohorts can also be brought together to enhance confidence in findings through replication^14^ and to address questions spanning different age periods or constructs.^15^

There are, however, a range of barriers to more fully realizing the potential gains of cross-cohort approaches. For example, discoverability of common and complementary data elements across cohorts can be hampered by different ways of describing and documenting data across studies. Restrictive data access protocols can limit the feasibility of analysing participant-level data from multiple cohorts. Working in siloes poses a barrier to achieving alignment between cohort measures and protocols, which limits opportunities for data alignment and pooling. Even where aligned data are available, cohort researchers can lack opportunities to connect across teams and with methods experts to develop necessary skills and knowledge.

In the Northern Hemisphere, initiatives have emerged to tackle these barriers by bringing child and adolescent cohort studies that capture a wide range of exposures and outcomes together in common platforms, enabling their use both independently and within cross-cohort designs. An example is Cohort and Longitudinal Studies Enhancement Resources (CLOSER),^16^ which was established in 2012 and now brings together 19 major longitudinal cohorts in the UK (14 child and adolescent). The EU Child Cohort Network similarly brings together 18 cohorts to allow investigations of early-life exposures and adult health outcomes.^17^ These platforms build on the important groundwork developed by consortia drawing studies together around specific conditions, such as the International Childhood Cancer Cohort Consortium (I4C) focused on childhood cancer,^5^ which have required the development of sophisticated governance models and methodological approaches to realize their objectives.^5^

### Origins and aims of LifeCourse

An equally rich history of early-life cohort studies in the Southern Hemisphere has been largely underrepresented in emerging platforms to date. In response, the Murdoch Children’s Research Institute (MCRI), located in Melbourne, Australia, established the LifeCourse initiative in 2013 (https://lifecourse.melbournechildrens.com), with the aim of bringing together the large hub of cohort studies that were hosted by or in collaboration with the MCRI at that time (representing over half of the total number of child and adolescent cohorts in the Australasian region). LifeCourse was established in partnership with the University of Melbourne Department of Paediatrics and the Royal Children’s Hospital (which, together with MCRI, comprises the Melbourne Children’s Campus) and drew on wide ranging collaborations via the participating cohorts and research teams. A small leadership committee oversees LifeCourse, supported by a project team, partnering with a statistical group that undertakes research in analytic methods for observational studies, and collaborating with investigators, project managers and data users across the cohorts.

The LifeCourse initiative now supports 33 studies in total, each of which is independently managed by their study team. This includes 22 active longitudinal cohorts that have metadata fully integrated into the LifeCourse platform and at a minimum data-sharing protocols in place that enable collaboration or data use beyond the primary study team (Table 1). Together these 22 core studies capture the development of >40 000 children and young people and their families (Table 1). They include traditional population-based prospective longitudinal cohort studies that are broadly representative, though participant retention and engagement of culturally and socially diverse populations, such as families from Aboriginal and Torres Strait Islander and refugee backgrounds, remain a key focus. They also include cohorts from randomized–controlled trials, largely in clinical populations, that have extended follow-up for analysis beyond the trial’s primary focus. Beyond the 22 core cohorts, LifeCourse continues to provide more basic support to an additional 11 studies (Supplementary Table S1, available as Supplementary data at *IJE* online) that were engaged early in the inception of the initiative and remain scientifically significant but reflect different study designs (e.g. tissue banks, cross-sectional surveys, cohort consortia) or have more restricted data access.

**Table 1:** Features of core longitudinal cohorts supported by the LifeCourse platform

Cohort name	*N*	Primary study type	Sampling frame	Year commenced	Age range (years)	Number of data collection waves	Study focus	Data acquisition				Protocol or illustrative reference
								Surveys	Bio samples	Imaging	Data linkage	
AQUA: Asking Questions about Alcohol in Pregnancy Study	2146	Cohort	Women attending one of seven antenatal clinics in 2011–12 who were <19 weeks’ pregnant with a single baby	2011	0–8	7	Alcohol consumption during pregnancy and health and development of index child at birth and over childhood	Y	Y	Y	Y	^37^
AREST CF (Australian Respiratory Early Surveillance Team for Cystic Fibrosis) Early Surveillance Program: Detection of early lung disease in cystic fibrosis	168	Case–control	The longitudinal inception cohort consists of children diagnosed with CF and recruited before 12 weeks of age. The repeated cross-sectional cohort consists of children diagnosed with CF aged 6 years and under. Control groups were also recruited	2006	0–8	Varies by case/control and sub-study involvement	Assessment, treatment and prevention of cystic fibrosis lung disease in young children	Y	Y	Y	–	^38^
ART Studies: Review of the health of adults conceived with and without Assisted Reproductive Technologies	Mothers: 1524, young adults: 1096	Case–control	ART mothers: traced from clinic database (Melbourne IVF and Monash IVF in Victoria, Australia). Non-ART mothers: population-based controls recruited by random digit dialling (households in Victoria, Australia). ART and non-ART young adults: approached with maternal consent	2008	18–35	2	Health and development of young adults born with and without assisted conception	Y	–	–	–	^39^
Australian Temperament Project (ATP)/Generation 3 (ATPG3)	ATP: 2443; ATPG3: 1167	Longitudinal cohort	A representative sample of families with a 4- to 8-month-old child attending maternal and child health centres across 20 local government areas in Victoria were recruited and followed every 2 years across childhood and adolescence and every 3 years across young adulthood. In 2012, the study expanded to a third generation by recruiting offspring born to original ATP participants and their partners	1983	ATP: 0–38, ATPG3: 0–12	15 (ATP), 5 (ATPG3)	Social-emotional development from infancy to adulthood, and transgenerational (pre-conception) determinants of infant mental health, attachment and wellbeing	Y	Y	Y	Y	^40^
Baby Biotics	167	RCT	Infants aged 0–3 months with infant colic. Recruitment was from a range of services widely used by and readily accessible to parents seeking medical advice regarding their crying babies in Melbourne, Australia, followed up at 3 years	2011	0–1	7	Effect of probiotic *Lactobacillus reuteri* on infant colic and maternal mental health and family functioning. Long-term outcomes of colic	Y	Y	–	–	^41^
Barwon Infant Study (BIS)	1074	Longitudinal cohort	Antenatal recruitment of eligible women from two hospitals in the Barwon region of Victoria (at 28 weeks’ gestation)	2010	0–11	12	An investigation into the early-life origins of a range of non-communicable diseases in the modern environment	Y	Y	Y	Y	^42^
Children’s Attention Project (CAP) and Neuroimaging of the Children’s Attention Project sub-study (NICAP)	497	Case–control	CAP: Grade 1 children with and without ADHD, recruited across 43 socio-economically diverse government primary schools across Melbourne, Australia. NICAP: Recruited from CAP cohort, with equal number of cases and controls	2011	7–13	5	ADHD with a range of outcomes: mental health, academic, family and child wellbeing, quality of life	Y	Y	Y	Y	^43^ ^,^ ^44^
Childhood to Adolescence Transition Study (CATS)	1239	Longitudinal cohort	All Grade 3 students (8–9 years of age) from a stratified cluster sample of schools in Melbourne, Australia were invited to take part	2012	8–17	10	The health and emotional development of children as they pass through puberty, the middle years of school and the transition to high school	Y	Y	Y	Y	
COBRA: Childhood Overweight BioRepository of Australia	500	Cohort	Presentation to the specialist weight management service at The Royal Children’s Hospital	2009	2–18	2	To develop a unique biorepository of data and biological samples from overweight and obese children	Y	Y	–	–	^45^
Early Language in Victoria Study (ELVS)	1910	Longitudinal cohort	Maternal and child health nurses approached all parents of babies aged 8–10 months within six local government areas of Melbourne, Australia	2003	0–20	14	Speech and language development from infancy to adulthood	Y	Y	Y	Y	^46^
HealthNuts	5300	Longitudinal cohort	12-month-old infants presenting for routine scheduled vaccination at local government-led immunization clinics across Melbourne, Australia	2007	1–15	4	Understanding the natural history and determinants of allergic disorders including food allergy, asthma, eczema and hay fever	Y	Y	Y	Y	^4^
International Youth Development Study (IYDS)	5769	Longitudinal cohort	A two-stage cluster sample design was used to recruit students in Victoria, Australia and Washington State, USA	2002	9–28	9	Risk and protective factors of healthy and problem behaviours in young people, and how differences in Australian and US cultures and schools affect youth development	Y	–	–	Y	^27^
Longitudinal Study of Australian Children’s Child Health CheckPoint (LSAC CheckPoint)	1874	Biophysical module within longitudinal cohort	LSAC had a two-stage clustered sampling design, randomly selecting 10% of all Australian postcodes (stratified by state and urban/rural), then children registered in Medicare Australia’s database and aged 3–19 months (B cohort) or 4–5 years old (K cohort). B cohort families who completed a Wave 6 interview were invited into CheckPoint	2004	0–18	One module within multi-wave study	LSAC is Australia’s largest and only nationally representative children’s longitudinal study. The cohorts are followed with a broad focus including health and development, education, family and parenting characteristics and socio-economic environment. LSAC’s Child Health CheckPoint is a one-off physical health and biospecimens module for the B cohort children and parents	Y	Y	Y	Y	^47^
Melbourne Infant Study: BCG for Allergy and Infection Reduction (MIS BAIR)	1272	RCT	Pregnant women attending participating antenatal clinics in Melbourne and Geelong were approached to participate. Pregnant women or mothers interested in joining the study but not being cared for at a study maternity site were also enrolled	2013	0–5	16	To assess the effect of neonatal BCG (tuberculosis) vaccination on clinical allergy and infection outcomes over the first 5 years of life	Y	Y	Y	–	^48^
Memory Maestros	Whole cohort: 1802. RCT: 452	RCT	Observational cohort: children in grade 1 classrooms from 44 schools in metropolitan Melbourne (Australia). RCT: those children from the observational cohort screened as having low working memory	2012	5–9	5	Development of working memory in children	Y	Y	–	Y	^49^
Mothers’ and Young People’s Study (MYPS)	1507	Longitudinal cohort	Prospective pregnancy cohort of first-time mothers and their first-born children recruited at six public hospitals in Melbourne	2003	0–18	15	Maternal mental health and wellbeing, child health and wellbeing from birth to age 18 years and intergenerational impacts of exposure to intimate-partner violence	Y	–	–	–	^50^
Peri/post-natal Epigenetic Twins Study (PETS)	250 twin pairs	Longitudinal cohort	Women attending multiple-pregnancy clinics at three Melbourne hospitals (Royal Women’s Hospital, Monash Medical Centre, Mercy Hospital for Women) who were at 18–22 weeks’ gestation	2007	0–11	7	Investigating whether epigenetic markers measured at birth and early life can provide clues to the causal links between intrauterine exposures influencing perinatal phenotype and the risk of chronic cardiometabolic and neurodevelopmental diseases later in life	Y	Y	Y	Y	^51^
right@home	736	RCT	Pregnant women attending antenatal clinics in select Victorian and Tasmanian regions with 2 or more of 10 risk factors	2013	0–7	17	Promoting equity in children’s early learning and development for families experiencing high levels of adversity	Y	Y	–	Y	^52^
Triple B: The Triple B Pregnancy Cohort Study (Bumps, Babies and Beyond)	1623	Longitudinal cohort	Women attending antenatal services attached to major hospitals, and specialist drug and alcohol antenatal services, in NSW and WA	2009	0–8	8	Effects of substance use and mental health during pregnancy in women and partners on infant development and family functioning	Y	Y	–	Y	^53^
Victorian Adolescent Health Cohort Study (VAHCS)/Victorian Intergenerational Health Cohort Study (VIHCS)	VAHCS: 2032; VIHCS: 1026	Longitudinal cohort	VAHCS: representative sample of mid-secondary school adolescents (aged 14–15 years) across Victoria (Australia) were selected using a two-stage cluster sampling procedure. VIHCS: all active members of VAHCS who reported having a child between the recruitment phases (2006 and 2014)	1992	VAHCS: 14–35, VIHCS: 0–8	11 (VAHCS), 4 (VIHCS)	Mental and physical health problems and risk behaviours in the adolescent-to-adulthood transition and the role of pre-conception factors in outcomes of the next generation	Y	Y	–	Y	^54^
VicCHILD: Victorian Childhood Hearing Impairment Longitudinal Databank	1000	Register with longitudinal data collection	Victorian children with permanent hearing loss. Since 2012, recruitment has been through the Victorian Infant Hearing Screening Program. Since 2016, additional recruitment has been through a paediatric hearing clinical service	2012	0–18.5	6	Advancing understanding of hearing loss	Y	Y	–	Y	^55^
VITALITY: Primary prevention of infant food allergy: an RCT of post-natal vitamin D supplementation	2681	RCT	Randomly selected council-run immunization sessions, maternal and child health nurse sessions, and online across Melbourne, Australia	2014	0–6	7	To assess the role of post-natal vitamin D supplementation for the prevention of infant food allergy, lower respiratory infections and eczema	Y	Y	–	Y	^56^

Y = Yes. ADHD, attention-deficit/hyperactivity disorder; RCT, randomized–controlled trial. For further details and updates since the time of submission, see https://lifecourse.melbournechildrens.com/cohorts/.

**Figure 1:**
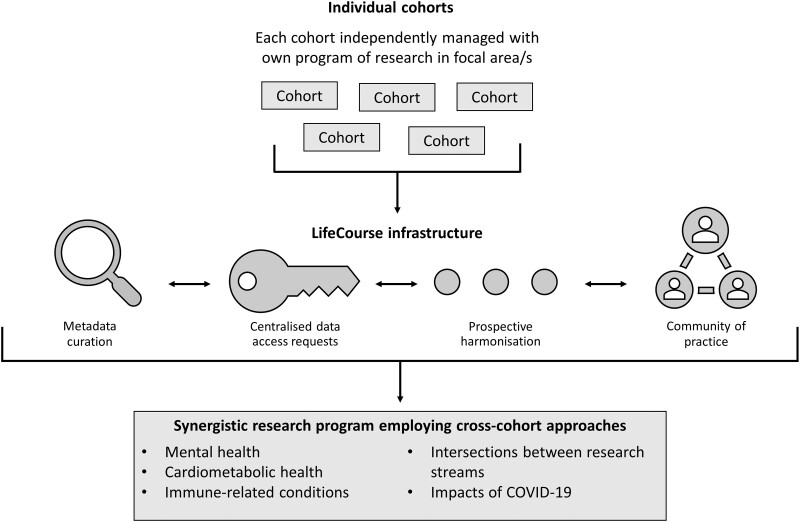
Overview of the LifeCourse initiative

LifeCourse aims to enable local and international researchers to capitalize on the availability of these extensive cohort data to advance understanding of health issues emerging over the life course. This includes an ongoing focus on addressing the major barriers to cross-cohort research, translated into four interrelated platform goals (Figure 1):

- Promoting data discoverability by curating browsable and searchable metadata, allowing researchers to easily identify relevant data available within and across cohorts.
- Facilitating data reuse by providing a central gateway for data access requests, which optimizes efficiency on the applicant side and ensures that all ethical and governance requirements are upheld for cohort custodians.
- Promoting prospective harmonization by providing guidance on common measurement tools and facilitating the collection of aligned cohort data around key initiatives.
- Creating opportunities for connection and synergy through a range of ‘meeting places’, including linking researchers to methodological expertise in the partnering statistical group.

The outcomes of these efforts include a growing programme of research focused on mental health, cardiometabolic health and immune-related conditions, strengthened through the use of cross-cohort methodologies enabled by the platform.

## Data collected

Across the 22 core cohorts (Table 1), data have been collected via surveys (e.g. web-based questionnaires), biosamples (e.g. blood), imaging (e.g. functional magnetic resonance imaging; fMRI), direct assessments (e.g. dental check), records abstraction (e.g. medical records) and data linkage (e.g. academic testing) (Table 1). There is considerable measurement consistency across outcomes and exposures relevant to focal areas of mental health (e.g. symptom inventories), cardiovascular health (e.g. obesity) and immune responses and related conditions (e.g. allergic diseases; Table 2). In 2020–2021, over half of the cohorts also rapidly adapted to collecting data on the direct (e.g. infection) and indirect (e.g. mental health) impacts of COVID-19.

**Table 2:** Data captured by core LifeCourse cohorts across key research streams

Cohort name	**Demographics** (e.g. gender, age, socio-economic position)	**Mental health** (e.g. symptom inventories, diagnosis)	**Cardiometabolic health** (e.g. risk and protective factors, direct assessments)	**Immune-related conditions** (e.g. allergies and eczema, inflammatory biomarkers)	**COVID-19 impacts** (e.g. infection, financial impacts)
AQUA: Asking Questions about Alcohol in Pregnancy Study	Y	Y	Y	–	–
AREST CF (Australian Respiratory Early Surveillance Team for Cystic Fibrosis) Early Surveillance Program: Detection of early lung disease in cystic fibrosis	Y	Y	Y	Y	–
ART Studies: Review of the health of adults conceived with and without Assisted Reproductive Technologies	Y	Y	Y	Y	–
Australian Temperament Project (ATP)/Generation 3 (ATPG3)	Y	Y	Y	Y	Y
Baby Biotics	Y	Y	–	Y	–
Barwon Infant Study (BIS)	Y	Y	Y	Y	Y
Children’s Attention Project (CAP) and Neuroimaging of the Children’s Attention Project sub-study (NICAP)	Y	Y	Y	–	–
Childhood to Adolescence Transition Study (CATS)	Y	Y	Y	Y	Y
COBRA: Childhood Overweight BioRepository of Australia	Y	Y	Y	Y	–
Early Language in Victoria Study (ELVS)	Y	Y	Y	–	Y
HealthNuts	Y	Y	Y	Y	Y
International Youth Development Study (IYDS)	Y	Y	Y	–	–
Longitudinal Study of Australian Children’s Child Health CheckPoint (LSAC CheckPoint)	Y	Y	Y	Y	–
Memory Maestros	Y	Y	Y	–	–
Melbourne Infant Study: BCG for Allergy and Infection Reduction (MIS BAIR)	Y	Y	Y	Y	Y
Mothers' and Young People's Study (MYPS)	Y	Y	Y	–	Y
Peri/post-natal Epigenetic Twins Study (PETS)	Y	Y	Y	Y	Y
right@home	Y	Y	Y	–	Y
Triple B: The Triple B Pregnancy Cohort Study (Bumps, Babies and Beyond)	Y	Y	Y	–	Y
Victorian Adolescent Health Cohort Study (VAHCS)/Victorian Intergenerational Health Cohort Study (VIHCS)	Y	Y	Y	–	Y
VicCHILD: Victorian Childhood Hearing Impairment Longitudinal Databank	Y	Y	–	–	Y
VITALITY: Primary prevention of infant food allergy: a randomized–controlled trial of post-natal vitamin D supplementation	Y	Y	Y	Y	Y

Y = Yes.

### Collation of metadata

To maximize discoverability of these participant data for researchers, the LifeCourse platform collates standardized study metadata for presentation on a publicly accessible website (https://lifecourse.melbournechildrens.com). Indexing rich metadata is critical to making cohort data Findable, Accessible, Interoperable and Reusable (the FAIR framework^18^) and aligns to the principle of Open Materials whereby details of a study’s design and measures are publicly accessible.^19^

**Figure 2:**
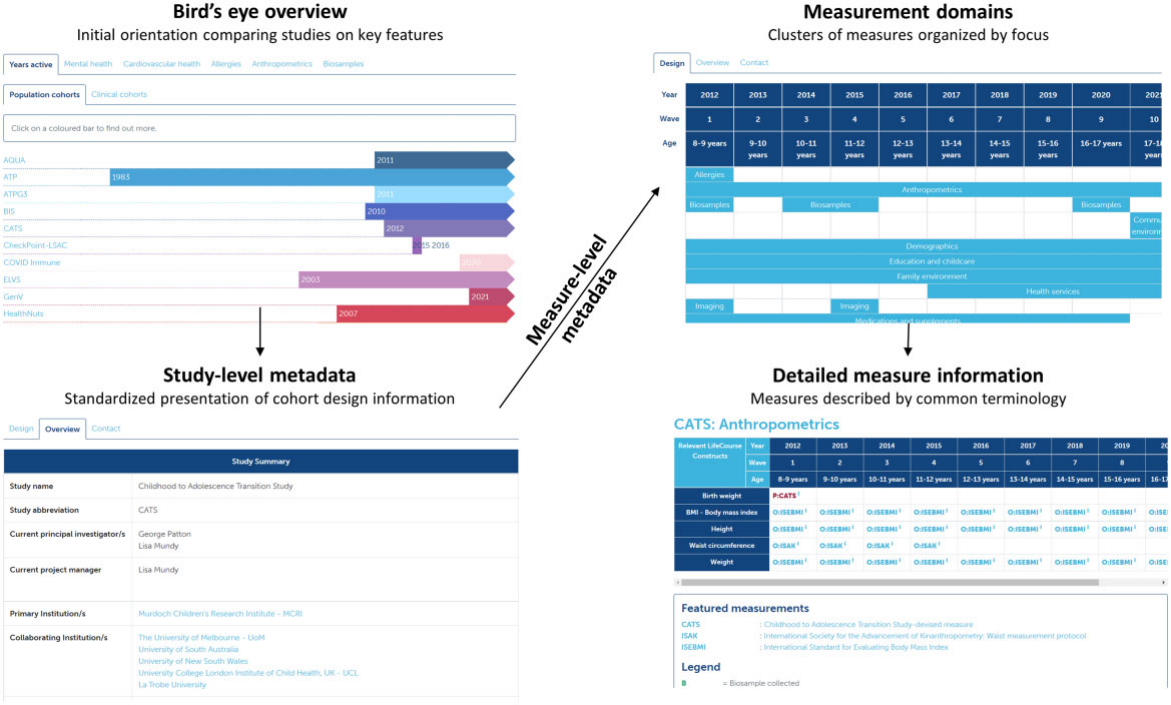
Standardized presentation of study-level and measure-level metadata on the LifeCourse website. Images taken with permission from https://lifecourse.melbournechildrens.com.

Study metadata are organized at several levels (Figure 2). At the highest level, description of key design features, such as the year established and number of participants, is provided in a standardized format. At a more detailed level, measures captured within each wave of data collection are described according to a common terminology (Systematized Nomenclature of Medicine; SNOMED^20^) and organized into content domains. SNOMED currently covers 80% of the concepts captured across LifeCourse cohorts and terms have been systematically developed for remaining gaps in areas such as education and childcare.^21^ Using this standardized system of description provides consistency with international standards and facilitates external comparisons of data availability.

### Prospective harmonization

Using identical measures and procedures across studies avoids the need to introduce assumptions about the equivalence of different measures or item sets during data pooling. However, this needs to be balanced with the potential consequences of overly prescriptive approaches.^22^ For example, many scales are not easily transferable across settings, such as from the clinical to community contexts.

LifeCourse facilitates the use of a common set of well-validated measures during the survey design phase where appropriate, such as through our measurement library (https://lifecourse.melbournechildrens.com/measurement-library/) while recognizing the need for study-specific approaches in many instances. In the COVID-19 context, for example, a number of cohorts collected aligned data from the CoRonavIruS health and Impact Survey (CRISIS)^23^ as well as specialized scales tailored to their own needs. This balance between common and study-specific measures enables cohorts to continue building their specialized research programmes while simultaneously facilitating cross-cohort research approaches to address shared questions. Harmonized protocols for the collection and analysis of biosamples have also proved advantageous, such as collaborative genotyping and epigenotyping of participants across cohorts.

## Data resource use

Each cohort has a vibrant individual programme of research aligning to their scientific agenda and benefitting from nuanced, study-specific data. Beyond the significant contributions of individual cohorts, a programme of research working across cohorts is increasingly emerging that addresses a shared interest in pathways leading to mental health, cardiometabolic health and immune-related conditions. The LifeCourse cohorts have collected particularly rich data in these domains (Table 2), allowing cross-cohort investigations within and at the intersections of these research streams, including in the COVID-19 context. This work is facilitated by LifeCourse’s efforts to enable data discoverability and access, promote prospective data harmonization and connect researchers across disciplinary boundaries. Below we outline illustrative examples of research using cross-cohort approaches in each of these key areas.

Both this cross-cohort research and that of individual cohorts are strengthened by the ongoing partnership between LifeCourse and the Melbourne Children’s campus Clinical Epidemiology and Biostatistics Unit (CEBU), which provides cohort researchers with access to cutting-edge methodological approaches. The CEBU methodological hub specializes in the development of novel methods and guidance in statistical areas that are key for longitudinal cohort studies, such as causal inference,^11^ as well as providing collaboration, open resources such as for analysis planning^24^ and training workshops. This is particularly relevant when using cross-cohort approaches, where new methodological challenges can arise.

### Mental health

The complex origins of mental health and illness emerge in the earliest periods of life and over two-thirds of core LifeCourse cohorts have tracked key exposures from infancy (Table 1). This has enabled investigation of the replicability of effects in the context of what are expected to be complex, multi-determined pathways to mental health and illness over long time spans.^25^ A particularly long temporal perspective is offered by two transgenerational cohorts tracking offspring of the original index child (Australian Temperament Project Generation 3 and Victorian Intergenerational Health Cohort Study). Strongly aligned protocols across these two studies have allowed intergenerational cycles of mental health to be explored in pooled data analyses.^26^ The availability of biosamples, such as co-ordinated extraction of genetic and epigenetic data from LifeCourse cohorts with well-aligned, repeated assessments of social-emotional development, is allowing investigation of the biological mechanisms that underpin these developmental pathways. International collaborations are progressing cross-national comparisons of intergenerational effects,^27^ the influence of differing school policies^28^ and the natural history of positive mental health and wellbeing.^29^

### Cardiometabolic health

Cardiometabolic disease as used herein refers to cardiovascular disease resulting from metabolic syndrome and its risk factors obesity and diabetes, and remains the leading cause of mortality worldwide. Following recent paradigm shifts, cardiometabolic disease is now conceptualized as a chronic inflammatory condition that develops from early life onwards, manifesting as progressive clinical disease predominantly in adulthood.^30^ LifeCourse provides an opportunity to investigate the early exposures and pre-clinical risk phenotypes for cardiometabolic disease, with in-depth phenotypic measures from birth to adulthood. Almost all LifeCourse cohorts contain some data relevant to the early origins of cardiometabolic health, such as (i) non-invasive assessments of pre-clinical large arterial vascular phenotypes (e.g. blood pressure, arterial stiffness and intima-media thickness, IMT); (ii) microvascular parameters; (iii) anthropometry and body composition; and (iv) circulating biomarkers of metabolic health and inflammation. Work is underway across LifeCourse and international cohorts to investigate how inflammation across the life course predicts these cardiometabolic phenotypes.

### Immune-related conditions

Immune dysregulation and inflammation are not only integral to common childhood conditions such as infection and allergic diseases, but also increasingly recognized as key mechanisms in the development of a range of adult non-communicable diseases, including mental health and cardiometabolic disease.^31^ Over half of the LifeCourse cohorts contain data relevant to immune health and these data are being used to understand how early-life exposures can drive immune dysregulation. For example, aligned data from the Barwon Infant Study (BIS) and the Longitudinal Study of Australian Children Child Health CheckPoint (LSAC CheckPoint) has demonstrated that children’s experiences of adversity relate to GlycA, an inflammatory biomarker, in both mid and late childhood.^32^ Allergy has also been a major focus of LifeCourse, which includes several internationally renowned cohorts established to investigate these conditions. For example, data from the HealthNuts and BIS cohorts have been used to compare allergy prevalence estimates across regional and metropolitan areas,^33^ whereas HealthNuts and LSAC CheckPoint have found inconsistent associations between caesarean delivery and asthma, and a negligible association with eczema.^34^

### COVID-19

The need to capture the direct and indirect effects of the COVID-19 pandemic for children and adolescents has provided further impetus for crossing traditional disciplinary boundaries. Longitudinal studies established prior to the pandemic are optimally positioned to show how COVID-19 may have impacted life-course trajectories.^35^ The COVID-Wellbeing working group has been formed to map pre-pandemic risk and resilience factors for mental health outcomes across the distinct populations of children and young people captured by LifeCourse cohorts. Collection of these data in a number of biomedically focused cohorts will allow examination of the interplay of social and biological factors. Findings will be used to inform the targeting of prevention and intervention efforts in the post-pandemic recovery period.

## Strengths and weaknesses

Beyond the quality, scope and richness of the underlying cohorts themselves, strengths of the LifeCourse initiative include the availability of richly described and structured cohort metadata, a common approach to data access requests and alignment of key data including for bioassays with many conducted in a single extraction on the same platform (e.g. metabolomics). This provides efficiency, comparability and feasibility in the use of these data, enhancing their value for promoting life-course health. This underlying infrastructure is further enhanced by partnering with methodologists and providing a range of other spaces fostering collaboration, driving the intellectual and human capital needed to make best use of these data.

Nevertheless, there are still a range of areas for further development. Making the process through which metadata are collated as simple and efficient as possible is key to improving accuracy and reducing time lags in the presentation of new metadata, such as in the COVID-19 context where many cohorts simultaneously pivoted to collecting data with time-critical applications. Raising the quality and comprehensiveness of the underpinning documentation in individual cohorts would further improve efficiency of metadata collation, which otherwise becomes progressively harder to remediate for long-running studies.^36^ Work is currently underway to develop best-practice data management templates and guides specific to the cohort context. The integration of required LifeCourse metadata fields into these templates will significantly enhance the ongoing sustainability of the platform.

Despite a central process for data access requests (outlined below), we do not yet have an integrated process through to data transfer. This is undertaken by the custodians of individual cohorts with varying processes and requirements aligning to their different governance structures and participant consents. We continue to work towards addressing ethics and governance barriers (e.g. promoting use of participant consents that allow appropriate data reuse) as well as technical infrastructure to support FAIR data provision. For example, the integrated data platform currently in development for the Generation Victoria ‘mega-cohort’ has the potential to support other studies in future, with enhanced features such as direct data browsing and a secure research environment for analyses. The ongoing efforts of LifeCourse to promote data discoverability and accessibility are critical to ensuing cohorts’ readiness to engage with such opportunities in future.

Finally, there are barriers to achieving these ambitions that are outside the immediate control (but perhaps in the sphere of influence) of LifeCourse and other such platforms. In moving towards Open Data, there must be mechanisms to acknowledge the teams responsible for data generation and to value this as an academic contribution without which the ensuing knowledge cannot accrue. The research community should be ambitious in addressing this fundamental issue rather than trying to curtail the efficient and ethical reuse of existing cohort data.^37^ Undertaking the types of cross-cohort research enabled by the platform typically requires more resourcing than single-cohort analyses, including in terms of biostatistical expertise, and so funding structures may also require review to appropriately resource this work and develop workforce capacity.

## Data resource access

### Community of practice

LifeCourse is designed to facilitate collaborations and engage new data users from within and beyond the Melbourne Children’s Campus. LifeCourse hosts a range of meeting places that provide space for new connections and collaborations to thrive (find out more at https://lifecourse.melbournechildrens.com and contact lifecourse@mcri.edu.au to join our mailing lists). Researchers not only benefit through synergistic research collaborations but can also deepen their collective expertise by sharing knowledge and experience about common issues.

### Centralized data access requests

To reduce logistical barriers to data access, LifeCourse acts as a liaison connecting data users and custodians. Applicants are invited to complete an initial enquiry through a central gateway (https://lifecourse.melbournechildrens.com/data-access/), requiring preliminary information on the team, primary research question and cohort/s of interest. LifeCourse confirms the in-principle feasibility of the request with the relevant cohort/s, after which an application is submitted with full details of the project and data and/or samples required. To overcome variations in governance structures across cohorts, cohort custodians retain decision-making responsibility and undertake the transfer of data and/or samples. Applications are assessed by cohorts for criteria such as (i) feasibility given the available data (e.g. quality issues with the data requested); (ii) consistency with ethical requirements (e.g. limits of participant consents); (iii) appropriateness for the purpose and strategic plans of the cohort (e.g. redundancy with research already underway); and (iv) scientific quality.

## Notes

The LifeCourse Cohort Investigators: Valerie Sung^1–3^; Emma Sciberras^4^; Sheena Reilly^5^; John W Toumbourou^4,6^; Kirsten P Perrett^1,2,7^; Catherine Marraffa^1,2,8^; Angela Guzys^1^; Jennifer J Koplin^1,2^; Stephanie J Brown^2,9^; Gehan Roberts^1,2,3^; Jon Quach^1,10^; Tim J. Silk^1,4^; Avihu Boneh^1,2^; Delyse Hutchinson^2,4,6,11^; Evelyne Muggli^1,2^; Sharon Lewis^2,12^

^1^Murdoch Children’s Research Institute, Melbourne, Australia; ^2^Department of Paediatrics, The University of Melbourne, Melbourne, Australia; ^3^Centre for Community Child Health, Royal Children’s Hospital, Melbourne, Australia; ^4^Centre for Social and Early Emotional Development, School of Psychology, Faculty of Health, Deakin University, Australia; ^5^Griffith University, Queensland, Australia; ^6^Centre for Adolescent Health, Murdoch Children’s Research Institute, Melbourne, Australia; ^7^Department of Allergy and Immunology, Royal Children’s Hospital, Melbourne, Australia; ^8^Department of Neurodevelopment and Disability, Royal Children’s Hospital, Melbourne, Australia; ^9^Intergenerational Health, Murdoch Children’s Research Institute, Melbourne, Australia; ^10^Melbourne Graduate School of Education, The University of Melbourne, Melbourne, Australia; ^11^National Drug and Alcohol Research Centre, Faculty of Medicine, University of New South Wales, Sydney, Australia; ^12^Reproductive Epidemiology, Murdoch Children’s Research Institute, Melbourne, Australia.

## Ethics approval

Ethics approvals for the studies described are managed by each individual study team, across a range of human research ethics committees.

## Author contributions

M.O’C. undertook primary drafting for most of the manuscript. M.M.-B. drafted and provided oversight for statistical/methodological components of the manuscript. C.O. and D.B. drafted specific sections of the manuscript and provided senior supervision for this work. All authors reviewed the manuscript and provided important intellectual content. The LifeCourse Cohort Investigators ensured accurate description of their cohort.

## Supplementary data

Supplementary data are available at *IJE* online.

## Funding

The Melbourne Children’s LifeCourse platform is funded by the Royal Children’s Hospital Foundation grant #2018–984, which includes support for M.O’C. M.M.-B. is the recipient of an Australian Research Council Discovery Early Career Researcher Award (project number DE190101326) funded by the Australian Government. S.G. is supported by Australian National Health and Medical Research Council (NHMRC) Practitioner Fellowship (1155290). C.O. is supported by an NHMRC Investigator Grant (APP1175086). D.B. is supported by an NHMRC Investigator Grant (1175744). M.W. is supported by an NHMRC Principal Research Fellowship (1160906). Research at the Murdoch Children’s Research Institute is supported by the Victorian Government’s Operational Infrastructure Program. The views reported in this paper are those of the authors only.

## Supplementary Material

dyac086_Supplementary_Data
