# Supplementary material for: Data Resource Profile: Melbourne Children’s LifeCourse initiative (LifeCourse)
Source: Int J Epidemiol. 2022 May 10;51(5):e229–44. doi: 10.1093/ije/dyac086 (PMC9557929; doi:10.1093/ije/dyac086)
Supplement: dyac086_Supplementary_Data [file dyac086_supplementary_data.pdf]

## Supplementary file

Table S1. Features of additional studies engaged with the LifeCourse platform.

| Study name | N | Study type | Data access beyond study team | Sampling frame | Year commenced | Age range captured (years) | Study focus | Protocol or illustrative paper |
|---|---|---|---|---|---|---|---|---|
| CCC: Children's Cancer Centre Tissue Bank | 1419 | Tissue bank | Y | New or existing patients attending the RCH with a suspected tumour, bone marrow disorder, Primary Immunodeficiency Disease or Neurofibromatosis, or a sibling, parent or relative of a patient described above. | 2014 | 0-25 | Cancer, Neurofibromatosis, bone marrow disorders and primary immunodeficiency disease. | |
| CLARITY: ChiLdhood Arthritis Risk factor Identification sTudY | 1470 | Cross-sectional | Y | Cases recruited from public or private clinics at RCH diagnosed with JIA by a paediatric rheumatologist before 16 years of age. Controls recruited from RCH Day Surgery Unit attending for minor surgical procedures. | 2008 | 0-19 | Identifying genomic and environmental disease risk factors for juvenile idiopathic arthritis. | [1] |
| I4C: International Childhood Cancer Cohort Consortium | All contributing cohorts >2000 participants | Cohort consortium | Y | Large population-based longitudinal birth cohorts with environmental measures in domains of interest for childhood cancers and the possibility of following children for childhood cancer. | 2005 | | To examine associations between environmental exposures and the incidence of childhood cancers by pooling prospective population data from one million pregnant mothers and their babies. | [2, 3] |
| Fontan: The Australian and New Zealand (ANZ) Fontan Registry | 1,574 | Register | Y | All individuals in Australia and New Zealand who have undergone the Fontan surgical procedure since it was first described in 1971. | 2009 | 18-53 | Health and wellbeing outcomes post-Fontan surgery. | [4] |
| PPOIT: Probiotic and Peanut Oral Immunotherapy Studies | 82 | RCT | N | Participants were recruited between December 2008 and March 2011 from the Royal Children's Hospital (Melbourne, Australia) Department of Allergy and Immunology outpatient clinics and through print media. | 2008 | 5-16 | Investigating the use of the probiotic Lactobacillus rhamnosus and peanut OIT to induce sustained unresponsiveness to peanut, in other words, a possible effective treatment for peanut allergy. | [5] |
| SchoolNuts | 9663 | Cross-sectional | N | Children aged 10-14 years (grade 5-8) and their parents recruited through randomly selected government, Catholic and independent primary and secondary schools (using stratified cluster sampling) within 80km of Melbourne's (Australia) Central Business District. | 2011 | 10-14 | Prevalence of challenge-proven food allergy and predictors of severe adverse reactions to food in early adolescence. | [6] |
| TAB: The Take A Breath Research Program | 194 | Cohort | N | Parents of children admitted to the Royal Children's Hospital (Melbourne, Australia), in | 2010 | 7-18 | Parent mental health and outcomes, following a serious illness or injury in their child. | [7] |

| Study name | N | Study type | Data access beyond study team | Sampling frame | Year commenced | Age range captured (years) | Study focus | Protocol or illustrative paper |
|---|---|---|---|---|---|---|---|---|
| | | | | the following departments: Oncology; Cardiology; Neurology; Intensive Care | | | | |
| VIBeS: Victorian Infant Brain Study | 227 | Case-control | N | 227 preterm children (born < 30 weeks' gestation or with birthweight < 1250 g) recruited from eligible admissions to the Royal Women's Hospital, Melbourne (Australia; control sample of 76 term children (born 37 to 42 weeks' gestation). | 2001 | 0-13 | Documenting the evolution of brain alterations and neurobehavioural impairments in premature infants. | [8] |
| VICIEM: A clinical and laboratory databank for patients with Inborn Errors of Metabolism | 1642 | Databank / Biobank | Y | Patients who presented at the RCH for treatment for an inherited metabolic disease (with records from 1970 to ongoing). | 2013 | 0-20+ | The natural history of inborn errors of metabolism (inherited metabolic diseases). | |
| Victorian Cerebral Palsy Register | 6,200 | Register | Y | Individuals who were born or have lived in Victoria, with a date of birth on or after 1st January 1970, whose condition fits the definition for cerebral palsy, i.e., a motor problem due to a lesion in the immature brain, which persists until after the age of 5 years and is non-progressive. The brain injury/abnormality must have occurred before the age of 2 years. | 1987 | 0-51 | The main aims of the VCPR are: to describe trends in the prevalence and clinical profile of cerebral palsy; to investigate causal pathways and identify potential avenues for prevention or amelioration of the condition; to identify specific groups of children/adults for further research; and, to contribute to other Australian and international datasets on cerebral palsy. | |
| VNTDR: Victorian Neural Tube Defects Registry | 235 | Register | N | Neural tube defect outpatient clinic RCH. | 2015 | 0-19 | To determine the best clinical care for children with spina bifida. | |

Note. Y=Yes, N=No or limited provision for data access at time of submission. RCH=Royal Children's Hospital. JIA=Juvenile idiopathic arthritis. RCT=Randomised Controlled Trial. For further details see: https://lifecourse.melbournechildrens.com/cohorts/.

**Supplemental references**


1. Ellis JA, Ponsonby A-L, Pezic A, et al. CLARITY–childhood arthritis risk factor identification sTudY. *Pediatric Rheumatology* 2012; **10**: 1-10.
2. Brown RC, Dwyer T, Kasten C, et al. Cohort Profile: The International Childhood Cancer Cohort Consortium (I4C). *International J Epidemiol* 2007; **36**: 724-30.
3. Tikellis G, Dwyer T, Paltiel O, et al. The International Childhood Cancer Cohort Consortium (I4C): a research platform of prospective cohorts for studying the aetiology of childhood cancers. *Paediatr Perinat Epidemiol* 2018; **32**: 568-83.
4. d'Udekem Y, Iyengar AJ, Galati JC, et al. Redefining expectations of long-term survival after the Fontan procedure: twenty-five years of follow-up from the entire population of Australia and New Zealand. *Circ* 2014; **130**: S32-S8.
5. Tang ML, Ponsonby A-L, Orsini F, et al. Administration of a probiotic with peanut oral immunotherapy: a randomized trial. *J Allergy Clin Immunol* 2015; **135**: 737-44. e8.
6. Sasaki M, Koplin JJ, Dharmage SC, et al. Prevalence of clinic-defined food allergy in early adolescence: The SchoolNuts study. *J Allergy Clin Immunol* 2018; **141**: 391-8. e4.
7. Muscara F, Burke K, McCarthy MC, et al. Parent distress reactions following a serious illness or injury in their child: a protocol paper for the take a breath cohort study. *BMC Psychiatry* 2015; **15**: 153.
8. Roberts G, Howard K, Spittle AJ, Brown NC, Anderson PJ, Doyle LW. Rates of early intervention services in very preterm children with developmental disabilities at age 2 years. *Journal Paediatr Child Health* 2008; **44**: 276-80.
